# Supplementary material for: Content-rich biological network constructed by mining PubMed abstracts
Source: BMC Bioinformatics. 2004 Oct 8;5:147. doi: 10.1186/1471-2105-5-147 (PMC528731; doi:10.1186/1471-2105-5-147)
Supplement: Additional File 2 — The original results of the above study (non-essential files are deleted to keep the file size under the limit set by BMC bioinformatics). [file 1471-2105-5-147-S2.bz2 › chilibotAdditionalFile2/dip30/464ID11259614E10491/html/CXCR3_CXCL9.html]

 


 **CXCR3** and **CXCL9** 
  
Found 192 abstracts in PubMed, retrieved 30.  
 

 What does Google say? 
 PDF only 
| .edu only 

---

**Interactive relationship** (e.g. stimulation, inhibition, etc)

**Neutral relationship**- Human microvascular endothelial cell line 1 HMEC 1, transfected with either the known  **CXCR3**  renamed  **CXCR3**  A or  **CXCR3**  B, bound  **CXCL9** , CXCL10, and CXCL11.  Ref: 12782716 J Exp Med, 2003
- Interferon gamma inducible 10 kd protein IP 10  [ **CXCR3** ]  and monokine induced by interferon gamma MIG  [ **CXCL9** ]  constituted 20% of 102 endotoxin induced clones identified in the preterm lamb lung.  Ref: 12598219 Am J Respir Crit Care Med, 2003
- AIM To assess the immunological role of human milk by analysing the concentrations of interferon gamma inducible protein of 10 kda IP 10  [ **CXCR3** ]  and monokine induced by interferon gamma MIG  [ **CXCL9** ]  in human milk from mothers of preterm and term infants.  Ref: 12856973 Acta Paediatr, 2003
- Interferon gamma inducible protein 10 IP10  [ **CXCR3** ]  and monokine induced by interferon gamma MIG  [ **CXCL9** ]  positive H& ; RS cells were detected in all 10 HL MC but only in 5 of 10 cases of HL NS and 2 of 9 cases of HL like ATLL.  Ref: 12866030 Int J CancerInt J Cancer, 2003
- Expression of CD4, CD8alpha, CD40, CD86, CD28, CD31 and vascular endothelial growth factor was not different between the two tumor groups but expression of CD40 ligand, CD80, NK1.1 and  **CXCR3**  was relatively lower in A11 Mig  [ **CXCL9** ]  tumors.  Ref: 12792744 Oncol Rep< MedlineTA>Oncol Rep, 2003
- The chemokine receptor  **CXCR3**  is predominantly expressed on T lymphocytes, and its agonists  **CXCL9** , CXCL10 and CXCL11 are IFN gamma inducible chemokines that promote Th1 responses.  Ref: 12884299 Eur J Immunol, 2003
- METHODS Using a semiquantitative polymerase chain reaction method, the authors analyzed the expression of chemokine mRNA coding for interferon IFN gamma induced protein 10 IP 10  [ **CXCR3** ]  and monokine induced by IFN gamma Mig  [ **CXCL9** ]  in a pool of human aortic endothelial cells.  Ref: 12698106 Transplantation, 2003
- DESIGN AND PATIENTS Immunocytochemistry and reverse transcriptase polymerase chain reaction RT PCR amplification were used to analyse the expression of monocyte chemoattractant protein MCP 1, RANTES regulated upon activation, normal T cell expressed and secreted, macrophage inflammatory protein MIP 1alpha, MIP 1beta, interferon IFN gamma inducible protein IP 10  [ **CXCR3** ]  and monokine induced by IFN gamma Mig  [ **CXCL9** ]  in thyroid tissue from patients with Hashimoto s thyroiditis n = 4, Graves disease n = 6 and nonautoimmune multinodular goitre n = 4.  Ref: 12864798 Clin Endocrinol (Oxf), 2003
- Interactions between IP 10  [ **CXCR3** ]  CXCL10, Mig  [ **CXCL9** ]   **CXCL9**  and I TAC CXCL11 and their shared receptor,  **CXCR3** , seem to be responsible not only for Th1 cell infiltration in acute allograft rejection and in proliferative GN, but also for mesangial cell proliferation typical of the latter condition.  Ref: 12508169 G Ital Nefrol< MedlineTA>G Ital Nefrol, 2003
- **CXCR3** , predominantly expressed on memory activated T cells, is a receptor for both IFN gamma inducible protein 10 CXC chemokine ligand CXCL 10 and monokine induced by IFN gamma  **CXCL9** .  Ref: 12538721 J Immunol, 2003
- An Alternatively Spliced Variant of  **CXCR3**  Mediates the Inhibition of Endothelial Cell Growth Induced by IP 10  [ **CXCR3** ] , Mig  [ **CXCL9** ] , and I TAC, and Acts as Functional Receptor for Platelet Factor 4.  Ref: 12782716 J Exp Med, 2003

**Non-interactive relationship** (e.g. studied together, co-existance, homology, etc.)

- RESULTS Expression of IP 10  [ **CXCR3** ] , Mig  [ **CXCL9** ] , I TAC, RANTES,  **CXCR3** , and CCR5, but not MCP 1 and IL 8, increased significantly in both grade 2 and grade 3 rejection P< ; =0.0096.  Ref: 12544874 Transplantation, 2003
- Assessment of cytokine production at the tumor site showed an interdependence of IFNgamma, MIG  [ **CXCL9** ]   **CXCL9**  and IP 10  [ **CXCR3** ]  CXCL10.  Ref: 12740040 Mol Cancer< MedlineTA>Mol Cancer, 2003
- Using an HBV transgenic mouse model, a reduction of hepatic infiltration and liver disease was achieved recently by administration of antibodies directed against the interferon gamma inducible chemokine Mig  [ **CXCL9** ]  and against IP 10  [ **CXCR3** ] .  Ref: 12794718 J Med Virol, 2003
- IP 10  [ **CXCR3** ]  and MIG  [ **CXCL9** ]  mRNA expression levels in cellular components of human milk were determined by RT PCR.  Ref: 12856973 Acta Paediatr, 2003
- The IP 10  [ **CXCR3** ]  and MIG  [ **CXCL9** ]  receptor  **CXCR3**  was detected in the bronchiolar epithelium of preterm lambs by immunostaining.  Ref: 12598219 Am J Respir Crit Care Med, 2003
- Potential molecular differences between early and late rejection grade > =2 episodes were examined by testing chemokine and receptor gene expression.METHODS Expression of inducible protein IP 10  [ **CXCR3** ] , monokine induced by IFN gamma Mig  [ **CXCL9** ] , interferon inducible T cell alpha chemoattractant I TAC, regulated on activation normal T cell expressed and secreted RANTES, and their receptors  **CXCR3**  and CCR5 was tested in 60 endomyocardial biopsies from 24 patients using quantitative Taqman real time polymerase chain reaction PCR.  Ref: 12829909 Transplantation, 2003
- Finally, TNFalpha IFNgamma was the most significant cytokine combination in leading to a pronounced upregulation in a comparable, time dependent manner of the production of chemokines IP 10  [ **CXCR3** ]  CXCL10, RANTES CCL5, MIG  [ **CXCL9** ]   **CXCL9** , MCP 1 CCL2, and IL 8 CXCL8.  Ref: 12555203 Glia, 2003
- RESULTS We assessed the importance of IFNgamma, IP 10  [ **CXCR3** ]  CXCL10 and MIG  [ **CXCL9** ]   **CXCL9**  in SLC CCL21 therapy.  Ref: 12740040 Mol Cancer< MedlineTA>Mol Cancer, 2003
- The chemokines  **CXCL9**  Mig  [ **CXCL9** ] , CXCL10 IP 10  [ **CXCR3** ] , and CXCL11 I TAC regulate lymphocyte chemotaxis, mediate vascular pericyte proliferation, and act as angiostatic agents, thus inhibiting tumor growth.  Ref: 12782716 J Exp Med, 2003
- Moreover, cytokines induced IL 15, IP 10  [ **CXCR3** ] , Mig  [ **CXCL9** ] , I TAC and MIP 3alpha protein accumulation in culture medium from human islets.  Ref: 12627325 Diabetologia, 2003
- In double staining,  **CXCR3**  Mig  [ **CXCL9** ]  coexpressing lymphoma cells were abundant in high MALT of the stomach and thyroid, but rare in other subtypes.  Ref: 12688353 Leuk Lymphoma, 2003
- CCR3 functional responses are regulated by both  **CXCR3**  and its ligands  **CXCL9** , CXCL10 and CXCL11.  Ref: 12884299 Eur J Immunol, 2003
- CONCLUSION We postulate that TFC may play a role in the pathogenesis of autoimmune thyroid disease as they are able to express the chemokines MIP 1alpha, MIP 1beta, MCP 1, RANTES, IP 10  [ **CXCR3** ]  and Mig  [ **CXCL9** ]  that would promote the infiltration of immune cells into the thyroid gland.  Ref: 12864798 Clin Endocrinol (Oxf), 2003
- Interferon gamma inducible chemokines  **CXCL9**  Mig  [ **CXCL9** ] , CXCL10 IP 10  [ **CXCR3** ] , CXCL11 I TAC, strongly associated to Th1 mediated immune responses, belong to the CXC sub family.  Ref: 12739748 J Endocrinol Invest, 2003
- Lesional keratinocytes also overexpressed the ligands of  **CXCR3** , namely, MIG  [ **CXCL9** ]   **CXCL9** , CXCL10, and I TAC CXCL11.  Ref: 12819030 Am J Pathol, 2003
- Remarkably, primary cultures of human microvascular endothelial cells, whose growth is inhibited by  **CXCL9** , CXCL10, CXCL11, and CXCL4, expressed  **CXCR3**  B, but not  **CXCR3**  A.  Ref: 12782716 J Exp Med, 2003
- Studies of cytokine and chemokine gene expression in PBMCs from eight patients revealed induction of IFN gamma, IP 10  [ **CXCR3** ] , and Mig  [ **CXCL9** ] .  Ref: 12684404 Clin Cancer Res, 2003
- Increased IP 10  [ **CXCR3** ]  and MIG  [ **CXCL9** ]  expression after intra amniotic endotoxin in preterm lamb lung.  Ref: 12598219 Am J Respir Crit Care Med, 2003
- Chemokines such as interleukin 8 IL 8, RANTES, IP 10  [ **CXCR3** ] , MIG  [ **CXCL9** ]  or lymphotactin, do not appear to induce fibrosis when over expressed in rodent lung.  Ref: 12570673 Curr Pharm Des, 2003
- Expression of IP 10  [ **CXCR3** ]  and MIG  [ **CXCL9** ]  genes and proteins in the milk cells as well as in mammary gland epithelial tissues was detected by RT PCR and immunohistochemistry.  Ref: 12856973 Acta Paediatr, 2003
- Both IP 10  [ **CXCR3** ]  and MIG  [ **CXCL9** ]  mRNA were induced 30 to 40 fold in the fetal lung at 1 to 2 days after IA endotoxin.  Ref: 12598219 Am J Respir Crit Care Med, 2003
- Expression of IP 10  [ **CXCR3** ]  and Mig  [ **CXCL9** ]  was not induced when alloantigen primed T cells from interferon gamma deficient recipients of C3H skin grafts were cultured with the endothelial cells.  Ref: 12605122 Transplantation, 2003
- A statistically significant increase of CXCL10 IP 10  [ **CXCR3** ]  and  **CXCL9**  Mig  [ **CXCL9** ]  expression, in thyroid tissue specimens obtained from subjects affected by Hashimoto s thyroiditis and recent onset Graves disease has been reported.  Ref: 12739748 J Endocrinol Invest, 2003
- CONCLUSION These findings indicate that the full potency of SLC CCL21 mediated anti tumor responses require in part the induction of IFNgamma, MIG  [ **CXCL9** ]   **CXCL9**  and IP 10  [ **CXCR3** ]  CXCL10.  Ref: 12740040 Mol Cancer< MedlineTA>Mol Cancer, 2003
- The CXC chemokines such as IL 8, IP10  [ **CXCR3** ] , Mig  [ **CXCL9** ] , SDF 1 alpha or CC chemokines such as MCP 1, MIP 1 alpha, eotaxin, RANTES have been frequently harvested from tumor tissues or the biological fluids of patients.  Ref: 12489485 Recenti Prog Med, 2002
- SLC CCL21 mediated anti tumor responses require IFNgamma, MIG  [ **CXCL9** ]   **CXCL9**  and IP 10  [ **CXCR3** ]  CXCL10.  Ref: 12740040 Mol Cancer< MedlineTA>Mol Cancer, 2002
- Our results indicate a tendency for low grade MALT to contain  **CXCR3**  Mig  [ **CXCL9** ]  lymphoma cells, high grade to contain  **CXCR3**  Mig  [ **CXCL9** ]  and DL to contain CRCR3 Mig  [ **CXCL9** ]  lymphoma cells.  Ref: 12688353 Leuk Lymphoma, 2003
- Moreover, in the kidneys of subjects suffering from chronic allograft nephropathy, IP 10  [ **CXCR3** ]  CXCL10, Mig  [ **CXCL9** ]   **CXCL9**  and I TAC CXCL11 have been found to be produced by and to act on the proxymal tubular epithelial cells, endothelial cells and smooth muscle vessel cells, suggesting their possible role in both the genesis of tubular atrophy and allograft artheriosclerosis.  Ref: 12508169 G Ital Nefrol< MedlineTA>G Ital Nefrol, 2003
- In contrast to endotoxin, IA tumor necrosis factor alpha or interleukin 1 alpha did not induce IP 10  [ **CXCR3** ]  or MIG  [ **CXCL9** ]  mRNA in the lung.  Ref: 12598219 Am J Respir Crit Care Med, 2003
- Expression of chemokine receptor  **CXCR3**  and its ligand, mig  [ **CXCL9** ] , in gastric and thyroid marginal zone lymphomas.  Ref: 12688353 Leuk Lymphoma, 2003
- BACKGROUND The goal of the current study was to test the ability of T cells to stimulate allogeneic endothelial cells to express chemokines, particularly the T cell recruiting factors monokine induced by interferon gamma Mig  [ **CXCL9** ]  and inducible protein IP 10  [ **CXCR3** ] .  Ref: 12605122 Transplantation, 2003
- This review discusses chemokine responses in relation to HSV infection of the cornea emphasizing the role of  **CXCR3**  chemokine signaling by the IFN gamma inducible ligands MIG  [ **CXCL9** ] , IP10  [ **CXCR3** ]  and I TAC and includes discussion of their potential role in immunopathology in the nervous system.  Ref: 12815542 Curr Eye Res, 2003
- IP 10  [ **CXCR3** ]  and MIG  [ **CXCL9** ]  are potent angiostatic chemokines that may contribute to lung injury and altered pulmonary vascular development in the preterm exposed to chorioamnionitis.  Ref: 12598219 Am J Respir Crit Care Med, 2003
- Our study was designed to investigate the expression of  **CXCR3**  and its ligand Mig  [ **CXCL9** ] , and their relationships in MALT using immunohistochemistry.  Ref: 12688353 Leuk Lymphoma, 2003
- 1 LARC CCL20 whose mRNA level increased rapidly at 3 h post challenge and then gradually decreased, 2 JE CCL2, MARC CCL7, MIP 1gamma CCL9, monocyte chemoattractant protein MCP 5 CCL12, ELC CCL19 and BRAK CXCL14 whose mRNA levels increased with time and reached the maximum at 6 9 h post challenge, 3 LIX CXCL5, Mig  [ **CXCL9** ]   **CXCL9**  and IP 10  [ **CXCR3** ]  CXCL10 whose mRNA levels increased gradually at least up to 12 h post challenge, and 4 SLC CCL21 whose mRNA level decreased gradually with time after challenge.  Ref: 12644322 Immunol Lett, 2003
- CONCLUSION The expression of IP 10  [ **CXCR3** ] , Mig  [ **CXCL9** ] , I TAC, RANTES,  **CXCR3** , and CCR5 in cardiac allografts significantly correlates with the presence and grade of acute rejection.  Ref: 12544874 Transplantation, 2003
- IP 10  [ **CXCR3** ]  CXCL10 and MIG  [ **CXCL9** ]   **CXCL9**  are T cell chemoattractants that have angiostatic properties.  Ref: 12598219 Am J Respir Crit Care Med, 2003
- There were significant correlations between MIG  [ **CXCL9** ]  and interferon gamma IFN gamma or IP 10  [ **CXCR3** ]  levels p <.  Ref: 12856973 Acta Paediatr, 2003
- Amplified fragments corresponding to KC, IP10  [ **CXCR3** ] , Mig  [ **CXCL9** ]  1, BCL, MIP 2, SLC, RANTES, MCP 1, C10, and Lptn were obtained, and confirmed by Southern blot and sequencing.  Ref: 12667214 Immunology, 2003
- IP 10  [ **CXCR3** ]  and MIG  [ **CXCL9** ]  protein expression in mammary gland tissues was analysed by immunohistochemistry.  Ref: 12856973 Acta Paediatr, 2003
- CONCLUSION IP 10  [ **CXCR3** ]  and MIG  [ **CXCL9** ]  in human milk, probably derived from milk cells and mammary gland epithelial cells, may contribute to the migration and activation of intestinal T lymphocytes to enhance mucosal immunity during the early neonatal period.  Ref: 12856973 Acta Paediatr, 2003
- IA endotoxin also caused a modest induction of IP 10  [ **CXCR3** ]  and MIG  [ **CXCL9** ]  mRNA in the jejunum, liver, and spleen.  Ref: 12598219 Am J Respir Crit Care Med, 2003
- We have previously shown that SLC CCL21 mediated anti tumor responses are accompanied by significant induction of IFNgamma and the CXC chemokines, monokine induced by IFNgamma MIG  [ **CXCL9** ]   **CXCL9**  and IFNgamma inducible protein 10 IP 10  [ **CXCR3** ]  CXCL10.  Ref: 12740040 Mol Cancer< MedlineTA>Mol Cancer, 2003
- RESULTS Alloantigen primed T cells induced endothelial cell expression of regulated on activation normal T cell expressed and secreted RANTES, IP 10  [ **CXCR3** ] , Mig  [ **CXCL9** ] , monocyte chemotactic protein 1, macrophage inflammatory protein 1alpha, and macrophage inflammatory protein 1beta within 5 hr of coculture.  Ref: 12605122 Transplantation, 2003
- However, 2 of 5 cases of HL like ATLL with EBV infection and 2 of 2 HL NS with EBV had IP10  [ **CXCR3** ]  MIG  [ **CXCL9** ]  positive H& ; RS cells.  Ref: 12866030 Int J CancerInt J Cancer, 2003
- RESULTS Significant amounts of IP 10  [ **CXCR3** ]  and MIG  [ **CXCL9** ]  were detected in human milk.  Ref: 12856973 Acta Paediatr, 2003
- RESULTS Expression of MCP 1, RANTES, MIP 1alpha, MIP 1beta, IP 10  [ **CXCR3** ]  and Mig  [ **CXCL9** ]  was demonstrated in all Hashimoto s and most Graves thyroid specimens but very little expression was detected in the nonautoimmune goitre samples.  Ref: 12864798 Clin Endocrinol (Oxf), 2003
- In contrast to CXCR4, the expression levels of the chemokine receptor,  **CXCR3** , and the migration of cells in response to  **CXCL9**  and CXCL10 increased after IL 2 treatment.  Ref: 12730102 Blood< MedlineTA>Blood, 2003
- METHODS IP 10  [ **CXCR3** ]  and MIG  [ **CXCL9** ]  levels of colostrum, early milk, mature milk and sera were measured by enzyme linked immunosorbent assay ELISA.  Ref: 12856973 Acta Paediatr, 2003
- The expression of interferon IFN gamma inducible protein IP 10  [ **CXCR3** ] , monokine induced by IFN gamma Mig  [ **CXCL9** ] , interferon inducible T cell alpha chemoattractant I TAC, regulated on activation normal T cell expressed and secreted RANTES, monocyte chemotactic protein MCP 1, interleukin IL 8, and the receptors  **CXCR3**  and CCR5 were tested using quantitative, real time polymerase chain reaction.  Ref: 12544874 Transplantation, 2003
- High levels of RANTES CCL5, MCP 1 CCL2, IP 10  [ **CXCR3** ]  CXCL10, and Mig  [ **CXCL9** ]   **CXCL9**  simultaneously with mononuclear cell infiltration in the lungs was found.  Ref: 12875978 Am J Pathol, 2003
- In vivo depletion of IP 10  [ **CXCR3** ]  CXCL10, MIG  [ **CXCL9** ]   **CXCL9**  or IFNgamma significantly reduced the anti tumor efficacy of SLC CCL21.  Ref: 12740040 Mol Cancer< MedlineTA>Mol Cancer, 2003
- The concentrations of IP 10  [ **CXCR3** ]  and MIG  [ **CXCL9** ]  in colostrum and early milk were significantly higher than those of sera from healthy controls or lactating mothers.  Ref: 12856973 Acta Paediatr, 2003
- RESULTS Mig  [ **CXCL9** ]  and IP 10  [ **CXCR3** ]  were expressed in human endothelial cells both after IFN gamma treatment and after PBMC co culture.  Ref: 12698106 Transplantation, 2003
- The biopsies were taken in the first 3 months or from the 9th to the 12th month following transplantation.RESULTS IP 10  [ **CXCR3** ] , Mig  [ **CXCL9** ] , RANTES,  **CXCR3** , and CCR5 expression levels were increased in the later versus earlier biopsies P< ; =0.01 despite no change in histologic rejection grade status.CONCLUSION These results demonstrate significantly increased expression of T cell chemoattractants in heart allografts during later rejection when compared with episodes occurring shortly after transplantation.  Ref: 12829909 Transplantation, 2003
